# In Vitro Susceptibility of Mastitis Pathogens Isolated from Clinical Mastitis Cases on Northern German Dairy Farms

**DOI:** 10.3390/vetsci7010010

**Published:** 2020-01-20

**Authors:** Josef Bolte, Yanchao Zhang, Nicole Wente, Volker Krömker

**Affiliations:** 1 Department of Microbiology, University of Applied Sciences and Arts, D-30453 Hannover, Germany; josef_bolte@gmx.de (J.B.); yanchao.zhang@hs-hannover.de (Y.Z.); Nicole.wente@hs-hannover.de (N.W.); 2 Department of Veterinary and Animal Sciences, Faculty of Health and Medical Sciences, University of Copenhagen, DK-1870 Frederiksberg C, Denmark

**Keywords:** bovine mastitis, antimicrobial susceptibility, minimal inhibitory concentration (MIC), staphylococci, streptococci, coliforms

## Abstract

The present research study investigated the susceptibility of common mastitis pathogens—obtained from clinical mastitis cases on 58 Northern German dairy farms—to routinely used antimicrobials. The broth microdilution method was used for detecting the Minimal Inhibitory Concentration (MIC) of *Streptococcus agalactiae* (n = 51), *Streptococcus dysgalactiae* (n = 54), *Streptococcus uberis* (n = 50), *Staphylococcus aureus* (n = 85), non-*aureus* staphylococci (n = 88), *Escherichia coli* (n = 54) and *Klebsiella* species (n = 52). Streptococci and staphylococci were tested against cefquinome, cefoperazone, cephapirin, penicillin, oxacillin, cloxacillin, amoxicillin/clavulanic acid and cefalexin/kanamycin. Besides cefquinome and amoxicillin/clavulanic acid, Gram-negative pathogens were examined for their susceptibility to marbofloxacin and sulfamethoxazole/trimethoprim. The examined *S. dysgalactiae* isolates exhibited the comparatively lowest MICs. *S. uberis* and *S. agalactiae* were inhibited at higher amoxicillin/clavulanic acid and cephapirin concentration levels, whereas *S. uberis* isolates additionally exhibited elevated cefquinome MICs. Most Gram-positive mastitis pathogens were inhibited at higher cloxacillin than oxacillin concentrations. The MICs of Gram-negative pathogens were higher than previously reported, whereby 7.4%, 5.6% and 11.1% of *E. coli* isolates had MICs above the highest concentrations tested for cefquinome, marbofloxacin and sulfamethoxazole/trimethoprim, respectively. Individual isolates showed MICs at comparatively higher concentrations, leading to the hypothesis that a certain amount of mastitis pathogens on German dairy farms might be resistant to frequently used antimicrobials.

## 1. Introduction

The pathogens predominantly associated with bovine mastitis in Germany are substantially staphylococci, streptococci and coliforms [1]. In general, clinical mastitis cases are treated using antimicrobial substances that are locally applied into the teat canal subsequent to the occurrence of clinical signs. The treatment of clinical bovine mastitis with antimicrobial agents is frequently induced without prior knowledge of causative agents and preferred use of antimicrobials covering a broad spectrum of pathogens. According to unpublished data provided by the German Consumer Research Company (GfK), udder injectors containing cephalosporins were used most frequently in the first quarter of 2017. Both cefoperazone and cefquinome, belonging to cephalosporins of the third- and fourth-generation, achieved a total market share of 39% in Germany. Other commonly used antimicrobial agents were the combinations cefalexin/kanamycin and amoxicillin/clavulanic acid with recorded market shares of 27% and 19%, respectively. However, an intramammary antimicrobial treatment of clinical mastitis induced by Gram-negative pathogens is frequently not recommended [2,3,4]. Moreover, the use of broad-spectrum antimicrobials is known to influence the development of resistance to a larger extent than narrow-spectrum antimicrobials [5]. Blanket antimicrobial therapy might thus not result in curing clinical mastitis, but rather increase the selective pressure on potentially present pathogens and is considered as an important aspect in developing resistance [5]. In Germany, the overall quantity of dispensed antimicrobials in 2017 decreased by 972.6 tons in comparison to 2011. While the group of penicillins was almost halved, third- and fourth-generation cephalosporins remained at a relatively constant level. The amount of dispensed fluorochinolones in 2017 increased by 6.1% compared to 2016, while the difference was even more pronounced in comparison to 2011 due to an increase of 20.1% [6]. Systematic resistance monitoring is a prerequisite for early detection of changes in the antimicrobial susceptibility of pathogens. Furthermore, this is also of major importance against the background of the detection of multi-drug-resistant pathogens on German dairy farms and the particular risk for humans due to the possibility of mutual exchange of resistances [7,8,9,10,11]. The aim of the present study was to establish an overview of the susceptibility of common mastitis pathogens obtained from clinical mastitis cases on dairy farms in Northern Germany with focus on routinely used antimicrobial agents. Therapy of subclinical mastitis cases is usually considered as not being economically beneficial, except for mastitis cases induced by contagious pathogens such as *Streptococcus agalactiae* [12]. Results of the current survey were further compared with previous German studies in order to ascertain changes in susceptibility patterns.

## 2. Material and Methods

### 2.1. Isolation and Identification of Pathogens

The isolates included in the current study were randomly selected from the strain collection of the University of Applied Sciences and Arts (Hannover, Germany). Examined mastitis pathogens were isolated from quarter foremilk samples of cows suffering from clinical mastitis. Milk sampling was performed between April 2013 and January 2018 on a total of 58 different Northern German dairy farms. Altogether, 434 isolates were included in the study, subdivided as follows: 85 *Staphylococcus* (*S.*) *aureus* (12 farms), 88 non-*aureus* staphylococci (NAS) (17 farms), 51 *Streptococcus* (*S.*) *agalactiae* (4 farms), 54 *Streptococcus* (*S.*) *dysgalactiae* (30 farms), 50 *Streptococcus* (*S.*) *uberis* (35 farms), 54 *Escherichia* (*E.*) *coli* (15 farms) and 52 *Klebsiella* species (15 farms). The number of at least 50 isolates, as well as the long period of time, enables a comprehensive statement about the susceptibilities of mastitis pathogens to commonly used antimicrobials in Northern Germany. The herd size of participating farms ranged from 55 to 2500 cows with an annual average milk yield between 7600 and 13,000 kg. At the time of milk sampling, the bulk milk somatic cell count (SCC) ranged from 88,000 to 334,000 cells/mL. According to the German Veterinary Association (DVG), an SCC of 100,000 cells/mL being present in the bulk tank milk is aimed for. An increase in SCC is due to an increased occurrence of inflammatory cells in the milk and thus reflects an important parameter for assessing the udder health status of a dairy herd. If the arithmetic mean value of the bulk tank milk sample is >300,000 cells/mL in two consecutive samples or a single sample exhibits >400,000 cells/mL, further measures are necessary for rapidly detecting animals affected by mastitis, including clinical investigations of individual animals as well as assessing secretions and cyto-microbiological analysis [13]. 

The collection of milk samples as well as the microbiological identification of pathogens were performed in accordance with the guidelines of the German Veterinary Medical Association [14]. For identifying present pathogens, 10 µL of each milk sample was plated on a quadrant of esculin blood agar (Oxoid, Germany). After 24 and 48 h of aerobic incubation at 37 °C, microbiological investigations were implemented at growing colonies. Blood agar plates were examined by colony morphology, haemolysis pattern and esculin hydrolysis. Gram staining and biochemical tests were performed for further differentiation of present pathogens. This included the examination of catalase activities (3% H_2_O_2_; Merck, Germany) as well as the performance of a clumping factor test (DiaMondiaL Staph Plus Kit, Sekisui Virotech, Germany)—for the differentiation of *S. aureus* and NAS. *S. aureus* isolates were confirmed by the detection of the specific *nuc* gene according to Saiful et al. (2006) [15]. Serological tests (DiaMondiaL Streptococcal Extraction Kit Sekisui Virotech, Germany) were used for identifying esculin-negative streptococci as *S. dysgalactiae* or *S. agalactiae* according to Lancefield group C and group B, respectively. A Rambach agar, modified as previously described by Watts et al. (1993) [16], served for distinguishing *S. uberis* from *Enterococcus* species. Gram-negative rods were verified by testing the activity of cytochrome oxidase C (Bactident oxidase, Merck, Germany) and the performance in an oxidative fermentative test (OF basal medium with the addition of D (+)-glucose-monohydrate, Merck, Germany). Coliform bacteria exhibiting glucose fermentation were further differentiated by Chromocult coliform agar (Merck, Germany), since *E. coli* appeared as blue colonies after incubation for 24 h at 37 °C. Concerning pink colonies growing on the Chromocult coliform agar, *Klebsiella* species could be distinguished from other coliforms by their lack of mobility during the oxidative fermentative test.

### 2.2. Antimicrobial Susceptibility Testing

Antimicrobial susceptibility testing was performed using the broth microdilution method in accordance with the DIN EN ISO 20776-1:2006 protocol [17]. The Minimal Inhibitory Concentration (MIC) is defined as the lowest antimicrobial concentration at which bacterial proliferation is no longer visually apparent and is therefore a parameter for a pathogen’s susceptibility to a certain antimicrobial agent. Within the present study, MIC values of mastitis pathogens were determined for commonly used antimicrobials in mastitis treatment in Germany. Therefore, polystyrene sterile microtiter plates (Greiner Bio One, Germany) contained antimicrobial concentrations in a two-fold dilution series. For investigating *Streptococcus* species, 5% of defibrinated horse blood (Oxoid, Germany) was added. Gram-positive pathogens were tested against cefquinome, cefoperazone, cephapirin, penicillin, cloxacillin, oxacillin, amoxicillin/clavulanic acid (4:1) and cefalexin/kanamycin (2:1) in concentrations ranging from 0.06 µg/mL to 32 µg/mL. The used combinations referred to the ratio that is present in commercial preparations. Besides cefquinome and amoxicillin/clavulanic acid (4:1) with concentrations ranging from 0.06 µg/mL to 32 µg/mL, marbofloxacin and sulfamethoxazole/trimethoprim (19:1) were tested against coliforms with concentrations ranging from 0.015 µg/mL to 8 µg/mL and 0.03 µg/mL to 16 µg/mL, respectively. *S. aureus* ATCC 29213 and *E. coli* ATCC 25922 (Leibniz Institute DSMZ—German Collection of Microorganisms and Cell Cultures, Germany) were used as reference strains. Inoculated microtiter plates were incubated at 37 °C for 16 to 20 h. Bacterial growth was determined due to the appearance of a visible turbidity, whereby the well without turbidity corresponded to the MIC of the respective pathogen.

## 3. Results 

The results of the MIC examination are presented in detail in the following Tables for Gram-positive (Table 1, Table 2, Table 3, Table 4 and Table 5) and Gram-negative (Table 6 and Table 7) mastitis pathogens, respectively. Besides the distribution of individual isolates according to determined MIC values, MIC_50_ and MIC_90_ values are given as well. The MIC_50_ and MIC_90_ values define the lowest concentration at which proliferation of at least 50% and 90% of the tested bacteria is inhibited, respectively [17]. 

### 3.1. Results of Streptococcus agalactiae (n = 51) Isolated from Northern German Dairy Farms

The distribution of 51 *S. agalactiae* isolates was dense for all antimicrobials investigated, extending over less than three dilution levels. MIC_50_ and MIC_90_ values corresponded to the same concentration level for cefoperazone (0.25 µg/mL), oxacillin (0.5 µg/mL), amoxicillin/clavulanic acid (0.5 µg/mL) and cefalexin/kanamycin (4 µg/mL). Fifty-one *S. agalactiae* isolates were inhibited at cefalexin/kanamycin MIC of 4 µg/mL, representing the highest MIC_50/90_ values among the tested antimicrobial agents. MIC_90_ of cefquinome, cephapirin, penicillin and cloxacillin were one dilution higher than their MIC_50_ values. The lowest MIC_50_ as well as MIC_90_ were determined as ≤0.06 µg/mL and 0.125 µg/mL for cefquinome and penicillin, respectively (Table 1). 

### 3.2. Results of Streptococcus dysgalactiae (n = 54) Isolated from Northern German Dairy Farms

Of the 54 *S. dysgalactiae* obtained from clinical mastitis, 49 and 51 isolates were inhibited at cefquinome and penicillin concentrations of ≤0.06 µg/mL, respectively, and thus represented the lowest MIC_50/90_ values. While cefoperazone MIC_50_ and MIC_90_ also corresponded to the same concentration level (0.25 µg/mL), MIC_90_ values of cephapirin, cloxacillin, oxacillin, amoxicillin/clavulanic acid and cefalexin/kanamycin were at least one dilution higher than their MIC_50_. Highest MIC values were determined for the combination of cefalexin/kanamycin (MIC_50/90_: 0.5/1 µg/mL). One single isolate was inhibited at comparatively higher concentrations of all antimicrobials tested (cefquinome: 2 µg/mL, cefoperazone: 16 µg/mL, cephapirin: 2 µg/mL, penicillin: 8 µg/mL, cloxacillin: 32 µg/mL, oxacillin: 32 µg/mL, amoxicillin/clavulanic acid: 4 µg/mL, cefalexin/kanamycin: 4 µg/mL). Additionally, two individual isolates were inhibited at a cefalexin/kanamycin concentration of 4 µg/mL (Table 2). 

### 3.3. Results of Streptococcus uberis (n = 50) Isolated from Northern German Dairy Farms

The widest distribution pattern of 50 *S. uberis* isolates was apparent for the first-generation cephalosporin cephapirin, ranging from ≤0.06 µg/mL to >32 µg/mL. Additionally, cephapirin MIC_50_ (0.25 µg/mL) and MIC_90_ (2 µg/mL) differed by three concentration levels, while oxacillin MIC_50/90_ was 2 µg/mL. With regard to the remaining antimicrobial agents, MIC_50_ and MIC_90_ values either differed by one (cefquinome, penicillin, cloxacillin, amoxicillin/clavulanic acid) or two (cefoperazone, cefalexin/kanamycin) concentration levels. The lowest MIC_50_ and MIC_90_ of *S. uberis* isolates were found for penicillin at concentrations of 0.125 µg/mL and 0.25 µg/mL, respectively. In contrast, the highest MIC_90_ values were investigated for cefoperazone as well as cloxacillin (4 µg/mL, respectively). Three individual *S. uberis* isolates showed cefalexin/kanamycin MIC values of ≥32 µg/mL, respectively, whereby two of these three isolates were also inhibited at a cloxacillin concentration of 32 µg/mL. Furthermore, one single isolate was not inhibited within tested cephapirin concentration ranges (MIC >32 µg/mL) (Table 3). 

### 3.4. Results of Staphylococcus aureus (n = 85) Isolated from Northern German Dairy Farms

The distribution pattern of *S. aureus* was dense in the case of cefalexin/kanamycin, referring to three concentrations levels. In contrast, the widest distribution pattern of 85 *S. aureus* isolates was determined for penicillin, ranging from ≤0.06 µg/mL to 32 µg/mL with MIC_50_ and MIC_90_ values of ≤0.06 µg/mL and 0.5 µg/mL, respectively. Since at least 90% of S. aureus isolates were inhibited at a cephapirin concentration of 0.5 µg/mL, the lowest MIC_90_ values were detected for both penicillin and cephapirin. With the exception of penicillin, MIC_90_ values were either one (cefquinome, cefoperazone, cefalexin/kanamycin) or two concentration levels (cephapirin, cloxacillin, oxacillin, amoxicillin/clavulanic acid) higher than their MIC_50_ values. Cefoperazone and the combination of cefalexin/kanamycin showed the highest MIC_50_ and MIC_90_ values at concentrations of 2 µg/mL and 4 µg/mL, respectively. Four *S. aureus* isolates were inhibited at cefoperazone concentrations of 16 µg/mL, whereby three bacterial isolates also had penicillin MICs of 16 µg/mL and one of them a penicillin MIC of 32 µg/mL (Table 4).

### 3.5. Results of Non-aureus Staphylococci (n = 88) Isolated from Northern German Dairy Farms

Regarding most antimicrobial agents tested against 88 NAS, the investigated MIC_90_ values were one dilution higher than respective MIC_50_ values, except for cefalexin/kanamycin (MIC_50/90_: 0.5 µg/mL, respectively) and penicillin (MIC_50/90_: 0.125/0.5 µg/mL). The highest MIC_50_ (4 µg/mL) and MIC_90_ (8 µg/mL) values were investigated for the third-generation cephalosporin cefoperazone. One single isolate exhibited MIC values of ≥16 µg/mL concerning all tested antimicrobial substances, except for the combination of cefalexin/kanamycin (0.5 µg/mL), while an additional one was inhibited at cefoperazone and penicillin concentrations of 16 µg/mL, respectively (Table 5). 

### 3.6. Results of Klebsiella Species (n = 52) Isolated from Northern German Dairy Farms

The highest MIC_50_ (4 µg/mL) and MIC_90_ (16 µg/mL) values of 52 *Klebsiella* species were determined for amoxicillin/clavulanic acid. Sulfamethoxazole/trimethoprim MIC_90_ values were three dilutions higher than MIC_50_ values, whereas MIC_90_ values of remaining antimicrobials were two dilutions higher than MIC_50_ values. One individual isolate was not inhibited at the highest concentrations of cefquinome (>32 µg/mL) and sulfamethoxazole/trimethoprim (>16 µg/mL), respectively (Table 6). 

### 3.7. Results of Escherichia coli (n = 54) Isolated from Northern German Dairy Farms

The MIC_90_ values of 54 *E. coli* isolates were two (cefquinome, amoxicillin/clavulanic acid) or four concentration levels higher (marbofloxacin) than their MIC_50_. The highest MIC_90_ value was determined for sulfamethoxazole/trimethoprim (>16 µg/mL), differing by at least six dilutions from MIC_50_. The lowest MIC_90_ was 0.5 µg/mL for cefquinome and marbofloxacin. Moreover, six (11.1%), three (5.6%) and four (7.4%) *E. coli* isolates exhibited MIC values above the highest concentration tested for sulfamethoxazole/trimethoprim (>16 µg/mL), marbofloxacin (>8 µg/mL) and cefquinome (>32 µg/mL), respectively (Table 7).

## 4. Discussion

Besides the strongly limited amount of available research data on the susceptibility of mastitis pathogens obtained from German dairy farms, the comparison is further complicated by various methods available for susceptibility testing. The performance of susceptibility testing in accordance with internationally approved guidelines as published by the German Institute for Standardization (referred to as DIN (Deutsches Institut für Normung e. V.)) ensures the high reproducibility of the test results. While the agar disk diffusion test represents a qualitative method, not only being inexpensive to implement and easy to use in practice, the broth microdilution method is a quantitative method considered as the “gold standard” for susceptibility testing due to its complexity and accuracy [18]. Results as well as derived interpretations differ with regard to the method used for susceptibility testing, this making direct comparison of these two methods quite difficult [19,20]. 

Within the present study, investigated coliforms did not differ in terms of inhibition by cefquinome and amoxicillin/clavulanic acid, whereas marbofloxacin and sulfamethoxazole/trimethoprim MIC_90_ of *E. coli* were at least two dilutions higher than those determined for *Klebsiella* species. More striking differences were apparent concerning cefoperazone MIC_90_ values of NAS (8 µg/mL) and *S. agalactiae,* as well as *S. dysgalactiae* (0.25 µg/mL, respectively), differing by five dilution levels. While *S. dysgalactiae* might be considered as susceptible to most tested antimicrobials due to the comparatively low MIC values, *S. uberis* frequently exhibited the highest MICs among examined Gram-positive pathogens. Even though oxacillin and cloxacillin are both semisynthetic β-lactams belonging to the group of isoxazolyl penicillins [21], most Gram-positive pathogens were inhibited at lower oxacillin concentrations.

Previous German studies completely inhibited *S. agalactiae* at a penicillin concentration of ≤0.06 µg/mL [22,23] and cefquinome concentrations of either ≤0.06 µg/mL [23] or ≤0.125 µg/mL [24]. Amoxicillin/clavulanic acid MIC_50/90_ corresponded to the same concentration in trials of Minst et al. (2012) (≤0.03 µg/mL) [22] and the national resistance monitoring program (0.125 µg/mL) [23]. Wente and colleagues (2016) investigated lower cephapirin MIC_50/90_ values (≤0.125 µg/mL) [24], whereas our results of cefoperazon and oxacillin MIC_90_ values are consistent with the latest resistance monitoring [23]. Therefore, previous studies obviously differed from our results concerning MIC values of cephapirin (MIC_50/90_: 0.25/0.5 µg/mL) and amoxicillin/clavulanic acid (MIC_50/90_: 0.5 µg/mL), whereas results of remaining antimicrobials were largely consistent. 

Our findings concerning *S. dysgalactiae* isolates are in agreement with former trials regarding cefquinome and penicillin, since at least 90% were inhibited at ≤0.015 µg/mL within various studies [22,23,25]. In contrast, Wente et al. (2016) examined a higher cefquinome MIC_90_ of 0.25 µg/mL, whereas cephapirin MIC values were in line with our study [24]. Oxacillin MIC_90_ determined within a previous study was 0.06 µg/mL [23] and thus one dilution lower than our study, whereas results of Tenhagen et al. (2006) were in agreement with our survey [25]. Amoxicillin/clavulanic acid MIC_50_ and MIC_90_ determined by Tenhagen et al. (2006) was 0.125 µg/mL, respectively [25], while other German findings (MIC_50/90_: ≤0.03 µg/mL) differed by at least two dilutions from our results [22,23].

MIC values of penicillin and oxacillin determined for *S. uberis* in the present study are in line with those in a former study [23]. Minst et al. (2012) investigated penicillin MIC_50_ and MIC_90_ of *S. uberis* at concentrations of 0.03 µg/mL and 0.125 µg/mL, respectively [22]. German studies varied widely regarding cefquinome MIC_90_ values of *S. uberis* that were determined as 0.125 µg/mL [25], 0.5 µg/mL [24] and 0.25 µg/mL [23], respectively. Similar differences were also apparent for amoxicillin/clavulanic acid MIC_90_ values: 0.25 µg/mL [25], 0.125 µg/mL [22], 0.5 µg/mL [23], respectively. 

The MIC_50/90_ of *S. aureus* agree regarding cefquinome [25,26], cefoperazone [26] and amoxicillin/clavulanic acid [25], whereas the MIC_90_ values of our trial were one dilution higher concerning oxacillin [25,26] and amoxicillin/clavulanic acid [26]. Wente and colleagues (2016) examined higher MICs of *S. aureus* regarding cefquinome (MIC_50/90_: 1/2 µg/mL) and cephapirin (MIC_50/90_: 0.25/1 µg/mL) [24]. Penicillin MIC values investigated within the latest resistance monitoring reveal distinct differences compared to our study. Even though MIC_50_ (0.03 µg/mL) predominantly agree with our results (≤0.06 µg/mL), MIC_90_ was determined as 16 µg/mL [26]. 

In comparison with the latest monitoring, results of NAS were in agreement with our study regarding MIC_50/90_ values of cefquinome and amoxicillin/clavulanic acid [26]. Wente et al. (2016) determined higher cefquinome MIC_50_ and MIC_90_ values of 1 µg/mL and 2 µg/mL, respectively [24]. Additionally, cephapirin MIC_90_ (1 µg/mL) was one dilution higher [24], whereas oxacillin and penicillin MIC_90_ [26] were in line with our research study. Results for cefoperazone also differed, since an MIC_50_ and an MIC_90_ of 1 and 4 µg/mL, respectively, were previously determined [26]. 

Susceptibility of *Klebsiella* species remained at a constant level within the latest resistance monitoring programs. MIC_90_ of our study differed by one to four dilutions, since the BVL (Bundesamt für Verbraucherschutz und Lebensmittelsicherheit) determined MIC_90_ of 0.06 µg/mL (cefquinome), 4 µg/mL (amoxicillin/clavulanic acid), 0.06 µg/mL (marbofloxacin) and 0.25 µg/mL (sulfamethoxazole/trimethoprim) [23,26].

Whereas cefquinome MIC_90_ of *E. coli* are in line with a former study [23], Wente and colleagues (2016) examined cefquinome MIC_90_ of 32 µg/mL [24]. Moreover, cefquinome MIC_90_ determined within the resistance monitoring also varied over the years: 0.125 µg/mL (2010), 8 µg/mL (2012), 0.125 µg/mL (2014) and 0.5 µg/mL (2016) [23,26,27,28]. Interestingly, similar strong fluctuations were apparent concerning marbofloxacin (0.03 µg/mL (2012), 0.5 µg/mL (2014), 0.06 µg/mL (2016)) and sulfamethoxazole/trimethoprim (0.25 µg/mL (2010, 2012), ≥64 µg/mL (2014, 2015)) [23,26,27,28]. 

The resistance monitoring achieved results from susceptibility examinations from various laboratories located in different German federal states. Minst et al. (2012) already detected higher resistance rates in districts with a high density of dairy farms and assumed that this might be due to a locally increased antimicrobial use [22]. The current study focused solely on mastitis pathogens obtained from dairy farms in Northern Germany, which is characterized as a particularly densely populated region of livestock [10,29]. Data on dispensed antimicrobials in veterinary medicine indicated a considerably higher amount in the North of Germany, especially in the federal state of Lower Saxony [6]. Although resistance of staphylococci to antimicrobials was frequently higher in regions with an above-average intensity of dairy cattle in Northern Germany, a previous research study concluded that the density of swine or cattle populations is not associated with the frequency of resistant bacteria in a region [29]. In contrast, Tenhagen et al. (2018) concluded that prevalence of MRSA seems to be related to the density of livestock and might thus be elevated in densely populated regions. Furthermore, Tenhagen and colleagues also found a positive correlation between prevalence of MRSA in bulk tank milk and the respective herd size. In this regard, 13.3% of milk samples obtained from herds with >80 cows were tested positive for MRSA, whereas the amount was 7.3% in herds having <80 cows [10]. The current study included mastitis pathogens from dairy herds with a herd size ranging from 55 to 2500 dairy cows. To investigate differences in susceptibilities in relation to herd size, the participating farms were divided into 30 small dairy farms (<92 cows) and 28 large ones (>92 cows). The limiting number of 92 cows was used as this number represents the current average herd size in the Northern German region [30]. Table 8 and Table 9 display the MIC values for Gram-positive and Gram-negative pathogens by differentiating between small and large dairy herds, respectively. *S. agalactiae* was isolated from small dairy farms only, so the results of this mastitis pathogen are not presented. 

The differences for both Gram-positive as well as Gram-negative pathogens almost exclusively refer to investigated MIC_90_ values. A higher MIC_50_ value of 1 µg/mL was detected for isolates from larger farms concerning the following bacteria-antimicrobial-combinations: *Klebsiella* species (sulfamethoxazole/trimethoprim), *S. aureus* (cefquinome) and NAS (oxacillin). Regarding the tested NAS isolates, no additional differences were apparent between small and large farms, whereas MIC_90_ values of *S. aureus* isolates from large dairy farms were one concentration level lower (penicillin: 0.25 µg/mL; amoxicillin/clavulanic acid: 0.5 µg/mL) or higher (cephapirin: 0.5 µg/mL; cefalexin/kanamycin: 4 µg/mL) than the respective values determined for the isolates from small dairy farms. In contrast, the investigated Streptococci from small farms were frequently inhibited at elevated antimicrobial MIC_90_ values. In the case of the tested *S. dysgalactiae* isolates, this was obvious for cefoperazone, cloxacillin, oxacillin and cefalexin/kanamycin; whereas *S. uberis* isolates from herds >92 cows showed higher MIC_90_ values for all antimicrobial agents included in the study. In this respect, cloxacillin MIC_90_ (16 µg/mL) and cefalexin/kanamycin MIC_90_ (8 µg/mL) of *S. uberis* from small dairy farms represented the greatest difference from the isolates obtained from large farms (oxacillin MIC_90_: 2 µg/mL; cefalexin/kanamycin MIC_90_: 1 µg/mL), which differ in three concentration levels. There are also noticeable differences in the MIC_90_ values of the antimicrobials tested against Gram-negative bacteria. *Klebsiella* species isolated from large farms were therefore inhibited at MIC_90_ values of cefquinome (0.5 µg/mL), marbofloxacin (0.125 µg/mL) and sulfamethoxazole/trimethoprim (4 µg/mL) that were one concentration level higher than respective MIC_90_ from *Klebsiella* isolates from small herds, respectively. The amoxicillin/clavulanic acid MIC_90_ values of investigated *Klebsiella* were determined as 1 µg/mL (small farm) and 16 µg/mL (large farm). When discussing the results of the *Klebsiella* species, the different number of bacterial isolates from small (n = 12) and large herds (n = 40) must be taken into consideration. Due to the substantially smaller quantity of isolates from small farms, a single isolate represented 8.3% of the total and consequently has a higher impact on MIC_50/90_ values which refer to the total amount of tested bacteria. Regarding *E. coli* isolates, the MIC_90_ values of cefquinome (0.5 µg/mL) and amoxicillin/clavulanic (16 µg/mL) were higher on small farms, whereas a comparatively higher MIC_90_ was detected for isolates tested against marbofloxacin (1 µg/mL) and sulfamethoxazole/trimethoprim (32 µg/mL). The difference of five sulfamethoxazole/trimethoprim concentration levels was the highest between *E. coli* from small (1 µg/mL) and large (32 µg/mL) farms. This was due to four (12.1%) *E. coli* isolates gained from large farms, that were not inhibited within the tested concentration range and thus had MICs of >16 µg/mL. The remaining 29 isolates (87.9%) obtained from large farms as well had MICs of concentrations ranging from 0.25 µg/mL to 2 µg/mL for sulfamethoxazole/trimethoprim. Taking everything into consideration, a general statement about the occurrence of less susceptible mastitis pathogens existing on larger dairy farms cannot be confirmed on the basis of this study. Even though antimicrobial MICs were higher in isolates from larger farms, especially in coliforms, the MIC_90_ of streptococci were exclusively higher in large dairy herds, while NAS showed no differences, except for oxacillin MIC_50_. However, more investigations including a greater bacterial number are needed before an accurate statement can be made. 

The susceptibility of Gram-positive mastitis pathogens obtained from dairy farms located in Northern Germany was previously described as favorable, whereas Gram-negative isolates exhibited varying degrees of resistance regarding all tested antimicrobials. Cephalosporins were considered to be effective against staphylococci and streptococci mastitis. However, penicillin represented the drug of choice for treating clinical mastitis induced by streptococci [31]. By establishing special guidelines, various countries aim to reduce the use of antimicrobials in order to counteract the development of resistance. This especially refers to those antimicrobial agents that were declared as “critically important” due to their major relevance in human medicine [32]. In Germany, obligatory guidelines have recently been introduced for the use of certain antimicrobial substances. The microbiological analysis of milk samples as well as susceptibility testing of present pathogens are thus required whenever use of third- and fourth-generation cephalosporins or fluorochinolones is considered for antimicrobial treatment [33]. In comparison to Germany, several Nordic countries such as Sweden, Norway, Denmark and Finland already created a common strategy for treating clinical mastitis several years ago. According to the jointly created “Nordic Guidelines for Mastitis Therapy” a general restrictive use of penicillin is pursued by an overall reduction in cephalosporins and quinolones as much as possible [12]. Chehabi et al. (2019) support the use of penicillin as the drug of first choice on Danish dairy farms based on its efficacy in in vitro susceptibility examinations of mastitis pathogens. Therefore, streptococci were classified as highly susceptible to penicillin, whereby results of *S. uberis* (MIC_50/90_: ≤0.06/0.25 µg/mL), *S. dysgalactiae* and *S. agalactiae* (MIC_50/90_: ≤0.06/≤0.06 µg/mL) were largely consistent with our results. Whereas penicillin MIC values of Danish NAS were ≤0.06 µg/mL (MIC_50_) and 0.5 µg/mL (MIC_90_), results of *S. aureus* were higher for penicillin MIC_90_ (2 µg/mL), while penicillin MIC_50_ was ≤0.06 µg/mL [34]. A lower penicillin MIC_90_ of Danish *S. aureus* was previously reported at concentrations of 0.25 µg/mL, this being identical with results of *S. aureus* isolates from Iceland and Switzerland (MIC_50/90_: ≤0.06/0.25 µg/mL). Penicillin MIC values of *S. aureus* from England, Finland, Ireland, Sweden and the United States were in accordance with our results detected at concentrations of ≤0.06 (MIC_50_) and 0.5 µg/mL (MIC_90_) [35]. Oxacillin MIC_50/90_ of *S. aureus* investigated in our study was determined at concentrations of 0.25 µg/mL and 1 µg/mL, respectively. In comparison to results of previous surveys from England, Finland, Iceland, Ireland, Norway, Sweden, Switzerland and the United States, the oxacillin MIC_90_ of *S. aureus* was also 1 µg/mL, while MIC_50_ was 0.5 µg/mL. *S. aureus* isolates from Denmark were inhibited at lower oxacillin concentrations (MIC_50/90_: 0.25/0.5 µg/mL) [35]. 

An interesting point is the difference in the MIC values of the isoxazolyl penicillins oxacillin and cloxacillin, which was detected for the majority of mastitis pathogens. Besides the problem of different methods used for susceptibility testing, comparison is also hampered by the use of different antimicrobial substances approved for mastitis treatment in various countries. The differences in the MIC values of oxacillin and cloxacillin indicate that a comparison of different antimicrobial agents belonging to the same antimicrobial group is inappropriate, such as comparing the third-generation cephalosporins cefoperazone (approved in Germany) and ceftiofur (approved in the United States). Most of the research studies only included oxacillin for detecting the in vitro efficacy of isoxazolyl penicillins against bacteria. An international study by de Jong et al. (2018) investigated the susceptibility of *S. aureus* and NAS against both oxacillin and oxacillin. Whereas NAS exhibited MIC_50_ values of cloxacillin and oxacillin at concentrations of 0.5 µg/mL, the MIC_90_ differed for cloxacillin (2 µg/mL) and oxacillin (1 µg/mL). In contrast, *S. aureus* was inhibited at cloxacillin MIC_50_ and MIC_90_ values of 0.25 µg/mL and 0.5 µg/mL, respectively, while MICs of oxacillin were one concentration level higher (MIC_50/90_: 0.5/1 µg/mL) [36]. Whereas the findings of NAS support the statement of higher cloxacillin MICs, the results of *S. aureus* did not support this hypothesis. De Jong et al. (2018) summarized the results of susceptibility testing from nine European countries: Belgium, the Czech Republic, Denmark, France, Germany, Italy, the Netherlands, Spain and the United Kingdom. Results of de Jong et al. (2018) were different to those of our study for *S. aureus* (amoxicillin/clavulanic MIC_50_: 0.125 µg/mL; cefquinome MIC_90_: 0.5 µg/mL) as well as for NAS (cephapirin MIC_50/90_: 0.125/0.25 µg/mL), whereby the MICs published by de Jong et al. (2018) were one concentration level lower. The remaining MIC_50/90_ of amoxicillin/clavulanic acid, cephapirin and cefquinome did not differ between our results and those of de Jong et al. (2018) [36]. Concerning *S. dysgalactiae* differences to our study were detected in MIC_90_ values only, as MIC_90_ values of ≤0.03 µg/mL (amoxicillin/clavulanic acid), 0.06 µg/mL (cephapirin) and 0.015 µg/mL (cefquinome) were previously described [36]. Compared to our result, the MICs of *S. uberis* were lower in the case of amoxicillin/clavulanic acid (MIC_50/90_: 0.25/0.5 µg/mL), cephapirin (MIC_90_: 0.5 µg/mL) and cefquinome (MIC_50/90_: 0.125/0.25 µg/mL) [36]. Both *Klebsiella* species and *E. coli* previously exhibited cefquinome MIC_50/90_ of 0.06/0.125 µg/mL and thus differed by one (MIC_50_) and two (MIC_90_) concentration levels from our trial [36]. Amoxicillin/clavulanic MIC_90_ published by de Jong and coworkers (2018) was 8 µg/mL regarding both *Klebsiella* species and *E. coli*. Moreover, 90% of *E. coli* isolates were inhibited at a marbofloxacin concentration of 0.06 µg/mL (MIC_90_), representing a three concentration level lower value compared to our study. Marbofloxacin MIC_50/90_ against *Klebsiella* species were formerly detected as 0.06 µg/mL and differed concerning both values from our investigations (MIC_50/90_: 0.03/0.125 µg/mL) [36]. As a conclusion of the comparison with previous German and international studies, it can be stated that staphylococci predominantly show the same susceptibility patterns to most antimicrobials. The values for *S. dysgalactiae* are mainly limited to low concentration levels and the *S. uberis* isolates in our study also deviates strongly from the former German and international results. The high MIC values compared to cefquinome could possibly be due to the increased use of these antimicrobials on German dairy farms. However, this is not supported in the case of other pathogens which did not show increased cefquinome MICs and the fact that MIC values of other antimicrobials (e.g., cephapirin) tested against *S. uberis* (Table 3) were also elevated, although they are not very relevant in mastitis therapy in Germany.

The favorable results of the Danish, Norwegian and Swedish mastitis pathogens could be due to a highly restrictive use of antimicrobials in these countries. Prudent use—in order to minimize the selective pressure—should therefore be a mandatory requirement whenever the use of antimicrobial agents is considered. In the context of antimicrobial reduction in mastitis treatment, the possibility of alternative treatment methods should be considered. An alternative treatment method for mastitis cases could be the use of bacteriophages as therapeutic agents (bacteriophage therapy). Bacteriophages (also called phages) are viruses that only target prokaryotic cells, mainly of one bacterial species, for inserting their DNA or RNA for propagation. After propagation, phage-coded enzymes induce the lysis of the bacterial cell (lytic propagation cycle) which leads to the release of next generation phages that are able to infect new host cells [37]. In a mouse model conducted by Capparelli et al. (2007), a bacteriophage therapy was able to achieve a complete reduction in the *S. aureus* in mice that were simultaneously infected with bacteria and phages [38]. In another study, *S. aureus* was previously isolated from dairy cows suffering from mastitis and used to induce mastitis in mice as well. Phage therapy was able to reduce the bacterial counts as well as the clinical degree of the disease. The authors considered the use of phages for an alternative therapy option for the treatment of bovine mastitis, but also mentioned the non-comparability of mastitis in mice and cows due to anatomical and physiological conditions [39]. The fact that lytic results investigated within a mouse model cannot directly be transferred to a dairy cow suffering from mastitis has already been demonstrated by Gill et al. (2006) [40]. In this former study, solely 16.7% (3/18) (*p* > 0.05) of the quarters infected with *S. aureus* achieved a bacteriological cure after a five-day treatment with an infusion of a high concentration of bacteriophage K. The authors assumed that an effective concentration of phages in the mammary gland of cattle could not be achieved in raw milk, among other things, due to an inhibition of the bacteriophages to bind to the host cell surface [40]. The investigations by Gill et al. (2006) refer to cows suffering from subclinical mastitis and, to the best of our knowledge, clinical trials are still lacking.

Moreover, a therapy option is the application of non-antimicrobial agents like products containing proteolytic enzymes such as chymotrypsin and trypsin [41] or homeopathic remedies [42]. The former was tested in cows suffering from mild to moderate clinical mastitis, while the latter was administered to chronically infected animals. In both studies, the authors concluded that the treatment could be considered as a possible alternative to antimicrobial therapy. Nevertheless, the efficacy of both products for treating severe clinical mastitis cases may be considered doubtful [41,42].

Another possibility for avoiding the extensive use of antimicrobials could be the application of vaccines. In several trials, the application of vaccines was able to cause a reduced severity of the clinical signs accompanied with mastitis cases caused by *S. uberis* and *E. coli* [43,44]. Furthermore, *S. uberis*-specific vaccines were beneficial concerning the reduction in somatic cell count and bacterial count [44]. While Schukken et al. (2014) achieved a reduction in both the incidence and prevalence of intramammary infections with staphylococci [45], previous observations by Tenhagen et al. (2001) were contrary. Therefore, the application of a herd-specific vaccination in order to prevent *S. aureus*-induced mastitis in heifers was not successful, since neither the prevalence of intramammary infections nor the incidence of clinical mastitis was significantly approved [46]. The broad range of alternative treatment options (bacteriophages, vaccination, enzymes, homeopathic agents) must be, as already recommended by the majority of authors, further researched in order to establish these methods as effective alternatives in mastitis therapy. Nevertheless, these points might be beneficial in the pursued aim of reducing the antimicrobial use on dairy farms in the future. 

The aim of the present study was rather to focus on mastitis pathogens having comparatively higher MIC values, so that success of an antimicrobial therapy might be predicted as doubtful. While in the case of most mastitis pathogens, this only referred to individual isolates, an evident proportion of *E. coli* isolates were not inhibited within tested concentration ranges (Table 7). This leads to the assumption that individual resistant isolates are present on German dairy farms. However, the final classification of a pathogen as resistant against a certain antimicrobial agent is performed by using clinical breakpoints. Regarding the performance of susceptibility testing, it is obligatory that the used methodology and interpretive criteria are performed following the same guidelines [20]. The Clinical and Laboratory Standards Institute (CLSI) established clinical breakpoints for only three antimicrobial agents referring to the indication of bovine mastitis: ceftiofur, penicillin/novobiocin and pirlimycin [47]. While ceftiofur and penicillin/novobiocin are even not approved for the intramammary application in Germany, pirlimycin is not relevant for mastitis treatment according to current market shares (unpublished data provided by the German Consumer Research Company (GfK)). Due to the lack of specific clinical breakpoints, identifying resistant pathogens is frequently based on breakpoints established for a different animal species, different indication or even established for human medicine. A transmission of these breakpoints is not appropriate and can cause misinterpretations of the results [20,48]. Moreover, in some trials, a classification of pathogens as susceptible and resistant did not result in the enhanced success of a therapy that was derived from the test results [49,50,51]. While the β-lactam antimicrobials are excreted to a high degree in the urine and thus reach elevated concentrations there, the clinical breakpoints related to urinary tract diseases were generally set higher for these antimicrobials [48]. Undoubtedly, the conditions of the urinary tract are not directly transferable to the mammary gland of a dairy cow and presumptive pathogens present in the bovine udder. In order to avoid the pitfalls associated with the use of improper clinical breakpoints, we focused rather on distribution patterns and MIC values. Changes in MIC values can therefore be detected by a comparison with former susceptibility trials, especially by considering regional differences. An assessment and discussion based on MIC values might thus be an appropriate alternative to strictly classifying pathogens as susceptible or resistant, supported by the fact that the statement is even questionable. However, the establishment of mastitis-specific clinical breakpoints is an indispensable tool for accurately assessing resistances and therapy decisions in future. 

## 5. Conclusions

In conclusion, determined MIC values of Gram-negative pathogens were frequently higher than previously reported. Among Gram-positive mastitis pathogens, differences in MIC values were striking for some pathogen-antimicrobial combinations. Compared to previous German studies, *S. uberis* and *S. agalactiae* exhibited higher amoxicillin/clavulanic acid and cephapirin MIC values, while *S. uberis* isolates were also inhibited at higher cefquinome concentrations. In contrast, penicillin MIC_90_ of *S. aureus* (0.5 µg/mL) investigated in our study was considerably lower than previously described (16 µg/mL). The majority of examined Gram-positive pathogens were inhibited at lower oxacillin than cloxacillin MIC values. Except for *S. agalactiae*, several mastitis isolates were inhibited at comparatively higher concentrations or not inhibited at the highest tested concentration. This leads to the hypothesis that a certain number of resistant isolates are present on Northern German dairy farms. Whereas the number usually referred to individual isolates, the quantity of presumptive resistant *E. coli* was higher. Nonetheless, an accurate estimation of resistance is necessarily related to establishing international clinical breakpoints referring to the indication of mastitis. 

## Figures and Tables

**Table 1:** Distribution of the Minimal Inhibitory Concentration (MIC) of *Streptococcus agalactiae* (n = 51) isolated from Northern German dairy farms ^1^.

Distribution of MIC (µg/mL)											
	≤0.06	0.125	0.25	0.5	1	2	4	8	16	32	>32
Cefquinome	*45*	**6**	**-**	**-**	**-**	**-**	**-**	**-**	**-**	**-**	**-**
Cefoperazone	**-**	1	***47***	3	**-**	**-**	**-**	**-**	**-**	**-**	**-**
Cephapirin	**-**	**-**	*36*	**15**	**-**	**-**	**-**	**-**	**-**	**-**	**-**
Penicillin	*36*	**15**	**-**	**-**	**-**	**-**	**-**	**-**	**-**	**-**	**-**
Cloxacillin	**-**	**-**	**-**	**-**	*35*	**16**	**-**	**-**	**-**	**-**	**-**
Oxacillin	**-**	**-**	**-**	***48***	3	**-**	**-**	**-**	**-**	**-**	**-**
Amox/clav ^2^	**-**	**-**	10	***40***	1	**-**	**-**	**-**	**-**	**-**	**-**
Cefa/kan ^3^	**-**	**-**	**-**	**-**	**-**	**-**	*51*	**-**	**-**	**-**	**-**

^1^ MIC_50_ and MIC_90_ are displayed in italics and bold, respectively. Bold and italic digits indicate that MIC_50_ and MIC_90_ were identical. ^2^ Amoxicillin/clavulanic acid. ^3^ Cefalexin/kanamycin.

**Table 2:** Distribution of the Minimal Inhibitory Concentration (MIC) of *Streptococcus dysgalactiae* (n = 54) isolated from Northern German dairy farms ^1^.

Distribution of MIC (µg/mL)											
	≤0.06	0.125	0.25	0.5	1	2	4	8	16	32	>32
Cefquinome	***49***	2	1	1	**-**	1	**-**	**-**	**-**	**-**	**-**
Cefoperazone	**-**	10	***40***	**-**	2	1	**-**	**-**	1	**-**	**-**
Cephapirin	*47*	**3**	1	2	**-**	1	**-**	**-**	**-**	**-**	**-**
Penicillin	***51***	1	1	**-**	**-**	**-**	**-**	1	**-**	**-**	**-**
Cloxacillin	10	*38*	**1**	1	1	1	1	**-**	**-**	1	**-**
Oxacillin	*40*	**9**	1	1	**-**	2	**-**	**-**	**-**	1	**-**
Amox/clav ^2^	*47*	**3**	2	1	**-**	**-**	1	**-**	**-**	**-**	**-**
Cefa/kan ^3^	**-**	**-**	6	*39*	**6**	**-**	3	**-**	**-**	**-**	**-**

^1^ MIC_50_ and MIC_90_ are displayed in italics and bold, respectively. Bold and italic digits indicate that MIC_50_ and MIC_90_ were identical. ^2^ Amoxicillin/clavulanic acid. ^3^ Cefalexin/kanamycin.

**Table 3:** Distribution of the Minimal Inhibitory Concentration (MIC) of *Streptococcus uberis* (n = 50) isolated from Northern German dairy farms ^1^.

Distribution of MIC (µg/mL)											
	≤0.06	0.125	0.25	0.5	1	2	4	8	16	32	>32
Cefquinome	4	1	13	*21*	**7**	3	**-**	1	**-**	**-**	**-**
Cefoperazone	**-**	**-**	5	**-**	*25*	14	**2**	3	1	**-**	**-**
Cephapirin	3	3	*27*	11	**-**	**2**	**-**	**-**	3	**-**	1
Penicillin	15	*21*	**10**	**-**	4	**-**	**-**	**-**	**-**	**-**	**-**
Cloxacillin	**-**	**-**	**-**	3	5	*31*	**7**	**-**	2	2	**-**
Oxacillin	**-**	1	2	1	8	***33***	1	4	**-**	**-**	**-**
Amox/clav ^2^	2	**-**	6	*33*	**9**	**-**	**-**	**-**	**-**	**-**	**-**
Cefa/kan ^3^	**-**	**-**	21	*21*	2	**1**	1	1	**-**	1	2

^1^ MIC_50_ and MIC_90_ are displayed in italics and bold, respectively. Bold and italic digits indicate that MIC_50_ and MIC_90_ were identical. ^2^ Amoxicillin/clavulanic acid. ^3^ Cefalexin/kanamycin.

**Table 4:** Distribution of the Minimal Inhibitory Concentration (MIC) of *Staphylococcus aureus* (n = 85) isolated from Northern German dairy farms ^1^.

Distribution of MIC (µg/mL)											
	≤0.06	0.125	0.25	0.5	1	2	4	8	16	32	>32
Cefquinome	**-**	**-**	4	*40*	**35**	5	1	**-**	**-**	**-**	**-**
Cefoperazone	**-**	**-**	**-**	19	12	*33*	**15**	2	4	**-**	**-**
Cephapirin	22	*31*	21	**6**	1	3	1	**-**	**-**	**-**	**-**
Penicillin	*64*	11	1	**3**	**-**	**-**	2	**-**	3	1	**-**
Cloxacillin	1	15	*30*	27	**8**	4	**-**	**-**	**-**	**-**	**-**
Oxacillin	**-**	13	*33*	22	**12**	1	1	3	**-**	**-**	**-**
Amox/clav ^2^	5	31	*27*	11	**5**	6	**-**	**-**	**-**	**-**	**-**
Cefa/kan ^3^	**-**	**-**	**-**	**-**	2	*56*	**27**	**-**	**-**	**-**	**-**

^1^ MIC_50_ and MIC_90_ are displayed in italics and bold, respectively. Bold and italic digits indicate that MIC_50_ and MIC_90_ were identical. ^2^ Amoxicillin/clavulanic acid. ^3^ Cefalexin/kanamycin.

**Table 5:** Distribution of the Minimal Inhibitory Concentration (MIC) of non-*aureus* staphylococci (n = 88) isolated from Northern German dairy farms ^1^.

Distribution of MIC (µg/mL)											
	≤0.06	0.125	0.25	0.5	1	2	4	8	16	32	>32
Cefquinome	4	1	11	*44*	**23**	3	1	**-**	1	**-**	**-**
Cefoperazone	3	1	**-**	3	9	21	*36*	**13**	1	1	**-**
Cephapirin	9	13	*45*	**16**	3	1	**-**	**-**	**-**	**-**	1
Penicillin	29	*19*	25	**9**	2	2	**-**	**-**	1	**-**	1
Cloxacillin	4	2	15	12	*36*	**14**	3	1	**-**	**-**	1
Oxacillin	6	3	17	*19*	**35**	4	3	**-**	**-**	**-**	1
Amox/clav ^2^	9	14	*27*	**34**	1	**-**	2	**-**	1	**-**	**-**
Cefa/kan ^3^	7	1	22	***54***	**-**	3	1	**-**	**-**	**-**	**-**

^1^ MIC_50_ and MIC_90_ are displayed in italics and bold, respectively. Bold and italic digits indicate that MIC_50_ and MIC_90_ were identical. ^2^ Amoxicillin/clavulanic acid. ^3^ Cefalexin/kanamycin.

**Table 6:** Distribution of the Minimal Inhibitory Concentration (MIC) of *Klebsiella* species (n = 52) isolated from Northern German dairy farms ^1^.

	Distribution of MIC (µg/mL)												
	≤0.015	0.03	0.06	0.125	0.25	0.5	1	2	4	8	16	32	>32
Cefquinome			16	*20*	10	**2**	1	1	1	**-**	**-**	**-**	1
Amox/clav ^2^			**-**	**-**	**-**	2	1	17	*21*	2	**4**	5	
Marbofloxacin	11	*22*	13	**1**	2	2	1	**-**	**-**	**-**			
Sulfa/trim ^3^		**-**	**-**	4	5	*17*	11	7	**7**	**-**	**-**	1	

^1^ MIC_50_ and MIC_90_ are displayed in italics and bold, respectively. Bold and italic digits indicate that MIC_50_ and MIC_90_ were identical. Concentrations in the gray fields were not tested. The figures in this field represent isolates that were not inhibited within the tested concentration range. ^2^ Amoxicillin/clavulanic acid. ^3^ Sulfamethoxazole/trimethoprim.

**Table 7:** Distribution of the Minimal Inhibitory Concentration (MIC) of *Escherichia coli* (n = 54) isolated from Northern German dairy farms ^1^.

	Distribution of MIC (µg/mL)												
	≤0.015	0.03	0.06	0.125	0.25	0.5	1	2	4	8	16	32	>32
Cefquinome			18	*20*	10	**1**	**-**	**-**	**-**	**-**	1	**-**	4
Amox/clav ^2^			**-**	**-**	**-**	**-**	**-**	8	*31*	9	**6**	**-**	
Marbofloxacin	9	*35*	1	**-**	1	**4**	1	**-**	**-**	**-**	3		
Sulfa/trim ^3^		**-**	**-**	**-**	2	*39*	6	1	**-**	**-**	**-**	**6**	

^1^ MIC_50_ and MIC_90_ are displayed in italics and bold, respectively. Bold and italic digits indicate that MIC_50_ and MIC_90_ were identical. Concentrations in the gray fields were not tested. The figures in this field represent isolates that were not inhibited within the tested concentration range. ^2^ Amoxicillin/clavulanic acid. ^3^ Sulfamethoxazole/trimethoprim.

**Table 8:** Comparison of MIC_50_ (µg/mL) and MIC_90_ (µg/mL) of Gram-positive mastitis pathogens obtained from small (<92 cows) and large (>92 cows) dairy herds.

		*S. dysgalactiae* ^1^		*S. uberis* ^2^		*S. aureus* ^3^		*NAS* ^4^	
**Agent**	**MIC_50/90_**	<92	>92	<92	>92	<92	>92	<92	>92
**Cefquinome**	**MIC_50_**	≤0.06	≤0.06	0.5	0.5	0.5	1	0.5	0.5
	**MIC_90_**	≤0.06	≤0.06	2	1	1	1	1	1
**Cefoperazone**	**MIC_50_**	0.25	0.25	1	1	2	2	4	4
	**MIC_90_**	1	0.25	8	2	4	4	8	8
**Cephapirin**	**MIC_50_**	≤0.06	≤0.06	0.25	0.25	0.125	0.125	0.25	0.25
	**MIC_90_**	0.125	0.125	2	0.5	0.25	0.5	0.5	0.5
**Penicillin**	**MIC_50_**	≤0.06	≤0.06	0.125	0.125	≤0.06	≤0.06	0.125	0.125
	**MIC_90_**	≤0.06	≤0.06	1	0.25	0.5	0.25	0.5	0.5
**Cloxacillin**	**MIC_50_**	0.125	0.125	2	2	0.25	0.25	1	1
	**MIC_90_**	0.5	0.125	16	2	1	1	2	2
**Oxacillin**	**MIC_50_**	≤0.06	≤0.06	2	2	0.25	0.25	0.5	1
	**MIC_90_**	0.25	0.125	8	2	1	1	1	1
**Amox/clav ^5^**	**MIC_50_**	≤0.06	≤0.06	0.5	0.5	0.25	0.25	0.25	0.25
	**MIC_90_**	0.125	0.125	1	0.5	1	0.5	0.5	0.5
**Cefa/kan ^6^**	**MIC_50_**	0.5	0.5	0.5	0.5	2	2	0.5	0.5
	**MIC_90_**	2	1	8	1	2	4	0.5	0.5

^1^*S. dysgalactiae*: n = 24 (<92 cows); n = 30 (>92 cows). ^2^*S. uberis*: n = 26 (<92 cows); n = 24 (>92 cows). ^3^*S. aureus*: n = 30 (<92 cows); n = 55 (>92 cows). ^4^ NAS: n = 42 (<92 cows); n = 46 (>92 cows). ^5^ Amoxicillin/clavulanic acid. ^6^ Cefalexin/kanamycin.

**Table 9:** Comparison of MIC_50_ (µg/mL) and MIC_90_ (µg/mL) of Gram-negative mastitis pathogens obtained from small (<92 cows) and large (>92 cows) dairy herds.

		*Klebsiella* Species ^1^		*E. coli* ^2^	
**Agent**	**MIC_50/90_**	<92	>92	<92	>92
**Cefquinome**	**MIC_50_**	0.125	0.125	0.125	0.125
	**MIC_90_**	0.25	0.5	0.5	0.25
**Amox/clav ^3^**	**MIC_50_**	4	4	4	4
	**MIC_90_**	1	16	16	8
**Marbofloxacin**	**MIC_50_**	0.03	0.03	0.03	0.03
	**MIC_90_**	0.06	0.125	0.5	1
**Sulfa/trim ^4^**	**MIC_50_**	0.5	1	0.5	0.5
	**MIC_90_**	2	4	1	32

^1^*Klebsiella* species: n = 12 (<92 cows); n = 40 (>92 cows). ^2^ *E. coli*: n = 21 (<92 cows); n = 33 (>92 cows). ^3^ Amoxicillin/clavulanic acid. ^4^ Sulfamethoxazole/trimethoprim.
